# Peroral endoscopic myotomy with fundoplication (POEM-F) for achalasia: Systematic review and meta-analysis

**DOI:** 10.1055/a-2536-8132

**Published:** 2025-03-14

**Authors:** Harishankar Gopakumar, Eugene Annor, Ishaan Vohra, Iman Andalib, Amy Tyberg, Avik Sarkar, Haroon Shahid, Mine Carames, Juan Carlos Carames, Giovanna Porfilio Gularte, Abed Al-Lehibi, Resheed Alkhiari, Amol Bapaye, Carlos Robles-Medranda, Michel Kahaleh

**Affiliations:** 1 24490 Gastroenterology and Hepatology, OSF Saint Joseph Medical Center, Bloomington, United States; 2 17120 Department of Internal Medicine, University of Illinois Chicago College of Medicine at Peoria, Peoria, United States; 3 17120 Department of Gastroenterology, University of Illinois Chicago College of Medicine at Peoria, Peoria, United States; 4 3673 Gastroenterology, Hackensack Meridian Hackensack University Medical Center, Hackensack, United States; 5 24263 Gastroenterology & Hepatology, Hackensack Meridian JFK University Medical Center, Edison, United States; 6 Gastroenterology, Santander Hospital, Bucaramanga, Colombia; 7 Gastroenterology, Instituto Misionero de Gastroenterología y Motilidad Digestiva, Posadas, Argentina; 8 37849 Gastroenterology and Hepatology, King Fahad Medical City, Riyadh, Saudi Arabia; 9 48032 Division of Gastroenterology, Department of Medicine, College of Medicine, King Saud University, Riyadh, Saudi Arabia; 10 Shivanand Desai Center for Digestive Disorders, Deenanath Mangeshkar Hospital and Research Center, Pune, India; 11 Gastroenterology, Instituto Ecuatoriano de Enfermedades Digestivas - IECED, Guayaquil, Ecuador; 12 Endoscopy, Omni Hospital, Guayaquil, Ecuador; 13 Gastroenterology, Foundation of Interventional and Therapeutic Endoscopy, New Brunswick, United States

**Keywords:** Endoscopy Upper GI Tract, Reflux disease, Motility / achalasia, Barrett's and adenocarcinoma, POEM

## Abstract

**Background and study aims:**

Gastroesophageal reflux (GER) and its long-term sequelae remain a concern following peroral endoscopic myotomy (POEM). POEM with fundoplication (POEM-F) is simultaneous fundoplication via pure natural orifice transluminal endoscopic surgery (NOTES). In this study, we evaluated the efficacy and safety of POEM-F in mitigating post-POEM GER.

**Methods:**

We performed a comprehensive electronic database search from January 2008 through June 2024 for studies evaluating outcomes of POEM-F performed for managing post-POEM GER. Pooled proportions were calculated using random-effects models. Heterogeneity was assessed using I ^2^ and Q statistics.

**Results:**

We included seven studies comprising 127 patients. Pooled technical success for POEM was 96.90%; 95% confidence interval [CI] 91.40–98.90. Pooled technical success of fundoplication was 92.30%; 95% CI 85.20–96.10. Clinical success in treating achalasia was 96.40%; 95% CI 90.70–98.60. Rate of wrap integrity on follow-up was 84.00%; 95% CI 66.00–93.40. Composite clinical success of POEM-F in mitigating post-POEM GER was 86.20%; 95% CI 73.80–93.20. Mean total procedure duration and fundoplication time was 115.74 minutes; 95% CI 103.53–126.96 and 55.28 minutes; 95% CI 47.35–63.20, respectively. The overall pooled major adverse events (AE) rate was 3.60%; 95% CI 1.40–9.40.

**Conclusions:**

POEM-F is an effective procedure with an acceptable AE rate in expert hands. It appears to offer clinical benefit in mitigating post-POEM GER. However, further standardization for evaluating clinically significant post-POEM GER and long-term benefit of POEM-F is warranted.

## Introduction

Peroral endoscopic myotomy (POEM) is now the preferred treatment approach for achalasia and certain other spastic esophageal motility disorders 1 2 3 4 5 . Following its introduction by Inoue et al, multiple studies have shown POEM to be minimally invasive, safe, and effective, with results comparable to laparoscopic Heller myotomy (LHM) 6 7 8 9 . However, symptomatic post-POEM gastroesophageal reflux (GER) and long-term adverse effects of prolonged distal esophageal acid exposure remain a major clinical concern 10 11 . Some studies report that up to half of patients undergoing POEM can develop GER within the first year, with 10% to 15% developing severe (Los Angeles grade C or D) reflux esophagitis 10 . Current evidence is also limited about whether post-POEM GER leads to long-term consequences such as long-segment Barrett's, dysplastic Barrett’s, or GER-related adenocarcinoma 11 . Myotomy performed during POEM or LHM results in a compromised anti-reflux barrier between the esophagus and the stomach, predisposing patients to future GER development 11 . However, unlike LHM, which is almost always accompanied by partial or full fundoplication, POEM and other endoscopic treatments, such as pneumatic dilation, are not. Identifying patient- or procedure-related risk factors that affect rates of post-POEM GER, such as obesity, length of full-thickness myotomy, a higher pre-POEM Eckhardt score, and previous pneumatic dilation have been proposed, but the evidence is currently inconclusive 12 13 .

In 2019, Inoue et al. described a pilot study evaluating endoscopic fundoplication performed in the same setting as POEM to reduce post-POEM GER 14 . This peroral endoscopic myotomy with fundoplication (POEM-F) procedure adds a simultaneous fundoplication via pure (non-laparoscopic) natural orifice transluminal endoscopic surgery (NOTES) to attenuate post-POEM acid exposure. In POEM-F, after an initial anterior myotomy is performed, transmural dissection is done to enter the peritoneum, following which a loop ligating device is anchored to the fundus of the stomach and the myotomy edge. This is then gradually tightened to form a wrap, simulating the surgical Dor partial anterior fundoplication 14 15 . Subsequent Asian studies and recent single-center case series from the United States also showed that POEM-F is feasible and safe with excellent short-term outcomes 15 16 17 18 19 20 21 .

Post-POEM GER can present a diagnostic challenge because it is often asymptomatic 11 . Conversely, most patients with esophageal motility disorders with reflux symptoms were found to have esophageal hypersensitivity to chemical and mechanical stimuli rather than pathologic acid exposure 22 . Furthermore, ambulatory pH monitoring could be unreliable in subjects with esophageal motility disorders. For example, Pond et al. reported that esophageal acidification identified on 24-hour pH-impedance monitoring off acid suppression in treated achalasia patients was more often related to acid fermentation rather than gastroesophageal reflux disease 22 . Surveillance endoscopies and long-term use of proton pump inhibitors (PPIs) have hence been recommended for all post-POEM patients. Long-term surveillance endoscopy and PPI use present challenges in terms of cost, potential adverse events (AEs), and non-compliance.

With increasing reports of POEM-F success in reducing post-POEM GER, we performed a systematic review and meta-analysis to pool all currently available data about clinical outcomes of POEM-F.

## Methods

### Search strategy

A literature search was conducted on June 17, 2024 of the electronic databases Medline (Ovid), EMBASE, and the Cochrane Library (Cochrane Central Register of Controlled Trials and Cochrane Database of Meta-Analysis). The search included studies from January 02, 2015 to the day of the search (June 17, 2024). Controlled vocabulary (MeSH, Emtree) and keywords were used. The keywords used were “Peroral endoscopic myotomy,” “POEM,” "Achalasia," "Gastroesophageal Reflux," "Fundoplication," and "GERD." References to reviewed articles were further scanned for additional studies. The retrieved studies were carefully examined to exclude potential duplicates or overlapping data. The searches had no language, regional, or publication type restriction. Studies from relevant references not found in the above search were considered for inclusion. All search results were saved to a citation management tool (EndNote) and the Bramer Method was used to remove duplicates. Preferred reporting items for systematic reviews and meta-analysis (PRISMA) statements were followed for reporting this review 23 .

### Study eligibility

Published studies were eligible if they reported single-session POEM with endoscopic fundoplication using the NOTES technique in managing achalasia. Endoscopic fundoplication performed using other techniques, such as transoral incisionless endoscopic fundoplication (TIF), was excluded. Articles were excluded if they were not in the English language. Studies in animal models, editorials, abstracts with incomplete data, case reports with inadequate data, and comments were excluded. Two authors (HG, EA) reviewed full-text articles and extracted data independently. These were then compared for accuracy. When data did not match, both reviewers reviewed the study a third time, and differences were resolved by mutual agreement or review by a third author (IV).

### Data extraction and quality assessment

Two authors (HG, EA) independently abstracted the following data into a standardized form: Study characteristics (primary author, period of study, year of publication, study design, and geographical location of the study population), baseline characteristics of the study population (number of patients enrolled and participant demographics), intervention details (total procedure time, fundoplication time and the experience of the operator[s]), outcomes (technical success of POEM, technical success of fundoplication, clinical success, and AEs). Differences were resolved by mutual agreement or review by a third author (IV). Quality of the included studies was evaluated using the modified Newcastle-Ottawa scale for non-randomized studies.

### Outcomes evaluated

Outcomes evaluated were technical success of POEM, technical success of endoscopic fundoplication using the NOTES procedure, clinical success based on parameters assessing for evidence of post-POEM GER, overall clinical success, total procedure time, fundoplication time, and AEs. Technical success of POEM and fundoplication were defined as successful completion of all the steps involved in the procedure. Clinical success of POEM was defined as resolution of dysphagia as determined by post-procedure Eckardt score ≤ 3. Overall clinical success of POEM-F in mitigating GER was defined as absence of clinically significant distal esophageal acid exposure based on a composite of various parameters used to evaluate for the same. These included integrity of fundoplication wrap on follow-up endoscopy, ambulatory pH measurement, endoscopic evidence of erosive esophagitis, need for regular PPI use, and measurement of distensibility index before and after POEM-F.

### Statistical analysis

We performed a random-effects meta-analysis to synthesize the data by pooling results of all the studies meeting the inclusion criterion that was identified in the literature search. Because this analysis involved outcomes of interventions in various settings spanning diverse centers internationally, we calculated pooled proportions with 95% confidence intervals (CIs) using a random-effects model to account for variations across studies. All treatment effect estimates are reported with 95% CIs for all outcomes. If the included studies did not provide mean and standard deviation, we estimated their values using the reported median and interquartile range (IQR), based on Luo et al 24 . Heterogeneity of studies was evaluated by Cochran’s Q test based on inverse variance weights and by calculating the *I ^2^* statistic. The Q-statistic provides a test of the null hypothesis that all studies in the analysis share a common effect size. If all studies shared the same true effect size, the expected value of Q would be equal to the degrees of freedom (number of studies minus 1). I ^2^ values of 0% to 39% were considered non-significant heterogeneity, 40% to 75% moderate heterogeneity, and 76% to 100% considerable heterogeneity. Forest plots were drawn to show the point estimates in each study in relation to the summary of the pooled estimate. The width of point estimates in the forest plots indicates the weight assigned to that study. Effects of publication and selection bias on the summary estimates were tested by the Egger bias indicator and Begg-Mazumdar bias indicators. Funnel plots were also constructed to assess potential publication bias using the standard error and diagnostic odds ratio. Descriptive statistics were used for demographic data and recorded as mean with standard deviation or frequency (percentage). Comprehensive Meta-Analysis (CMA) software, Version 4, was utilized to perform statistical analysis for this study.

## Results

**Fig. 1:**
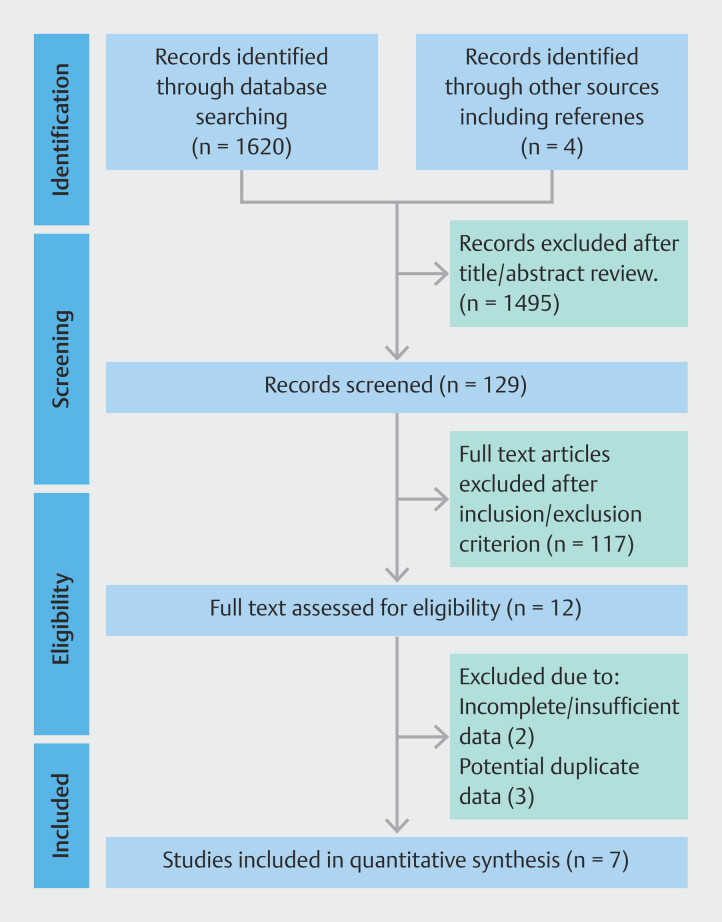
Study flow diagram according to the PRISMA guidelines 23 .

The initial search identified 1620 studies, of which relevant articles were reviewed after title and abstract evaluation. Data were extracted from seven studies comprising 127 patients that met inclusion criteria. Five studies were available in full-text format 14 15 16 18 19 , whereas two were available only as published abstracts 20 21 . Fig. 1 shows the search strategy according to PRISMA guidelines. Characteristics of the included studies, demographics, and patient characteristics are shown in Table 1 . The quality of studies was good as evaluated using the modified Newcastle-Ottawa scale for observational studies, as shown in Table 2 . All the pooled estimates given are estimates calculated by the random-effects model. Table 3 shows the main outcomes reported from individual studies.

The total sample size was 127 patients, with 41.73% females. Mean patient age was years 44.51 years (SD = 7.40). Most patients had Type II achalasia (78%), followed by Type I (16.53%). Mean pre-procedure Eckhardt score was 7.38 (SD = 1.19). All the studies used an anterior approach for POEM. Mean total procedure duration was 115.74 minutes; 95% CI 103.53–126.96 and mean fundoplication time was 55.28 minutes; 95% CI 47.35–63.20. Mean post-procedure hospital stay was 2.54 days 95% CI 1.08–4.00. Mean follow-up duration was 4.72 months (range 1–12).

**Table:** **Table 1** Study design, details, and patient demographics.

**Author, year**	**Study design, location**	**Patients (n)**	**Males (n)**	**Mean age (years)**	**Achalasia type I/II/III/other**	**Mean pre-procedure Eckhardt score**	**Mean follow-up duration (months)**
**Inoue et al, 2019**	Single-center prospective, Japan	21	10	45.40	13/5/1/2	5.7	2
**Bapaye et al, 2021**	Single-center retrospective, India	25	13	40.10	1/23/1/0	8.21	12
**Patil et al, 2021**	Single-center retrospective, India	20	14	32.00	2/18/0/0	7.2	3
**Mandavdhare et al, 2022**	Single-center retrospective, India	3	3	41.30	3/0/0/0	NA	1
**Shrigiriwar et al, 2023**	Single-center retrospective, USA	6	5	50.80	0/6/0/0	8.8	1
**Andalib et al, 2024**	Multi-center retrospective, Latin America	21	10	54.00	2/19/0/0	NA	11
**Fayyaz et al. 2024**	Single-center retrospective, USA	31	19	48.00	0/28/0/3	7	3.1
NA, not available.							

**Table:** **Table 2** Modified Newcastle Ottawa scale evaluating the quality of included observational studies.

**Author, year**	**Representativeness of exposed cohort**	**Selection of non-exposed cohort**	**Ascertainment of exposure**	**Outcome of interest not present at study start**	**Comparability**	**Assessment of outcome**	**Was follow-up long enough for outcome to occur**	**Adequacy of follow up**	**Quality score**	**Quality**
**Inoue et al, 2019**	*	NA	*	*	NA	*	*	*	6	High
**Bapaye et al, 2021**	*	NA	*	*	NA	*	*	*	6	High
**Patil et al, 2021**	*	NA	*	*	NA	*	*	*	6	High
**Mandavdhare et al, 2022**	*	NA	*	*	NA	*	*		5	High
**Shrigiriwar et al, 2023**	*	NA	*	*	NA	*	*		5	High
**Andalib et al, 2024**	*	NA	*	*	NA	*	*	*	6	High
**Fayyaz et al. 2024**	*	NA	*	*	NA	*	*	*	6	High
*Criterion in the corresponding column was satisfied by the study. NA, not applicable.										

**Table:** **Table 3** Study outcomes.

**Author, year**	**Patients (n)**	**Technical success fundoplication (n)**	**Wrap integrity (n)**	**Abnormal ambulatory pH testing (n)**	**Presence of LA Grade B, C or D esophagitis**	**Regular PPI use (n)**	**Positive GERD Questionnaire (n)**	**Overall Clinical Success of POEM-F (n)**
**Inoue et al., 2019**	21	21	20	NR	NR	NR	NR	20
**Bapaye et al., 2021**	25	23	19	2	0	0	1	21
**Patil et al., 2021**	20	17	10	7	4	5	NR	10
**Mandavdhare et al., 2022**	3	3	3	NR	NR	NR	NR	3
**Shrigiriwar et al., 2023**	6	6	NR	NR	NR	6	0	6
**Andalib et al., 2024**	21	21	NR	2	NR	NR	NR	19
**Fayyaz et al., 2024**	31	30	17	4	3	NR	NR	26
GERD, gastroesophageal reflux disease; LA Grade, Los Angeles grade; NR, not reported; POEM, peroral endoscopic myotomy.								

### Technical and clinical success

**Fig. 2:**
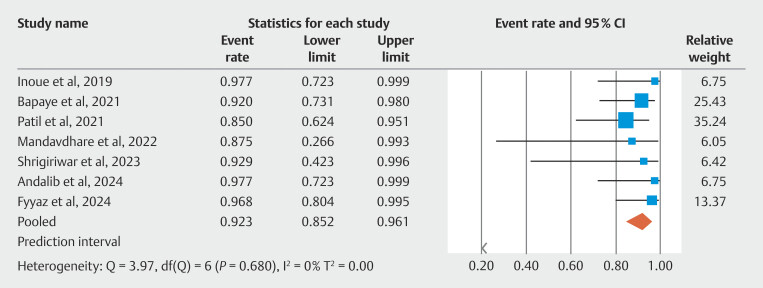
Forest plot showing individual study estimates and pooled estimate for technical success of fundoplication.

**Fig. 3:**
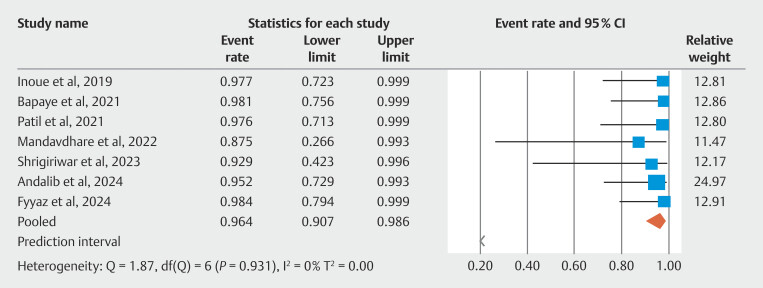
Forest plot showing individual study estimates and pooled estimate for clinical success of POEM in treating achalasia.

**Fig. 4:**
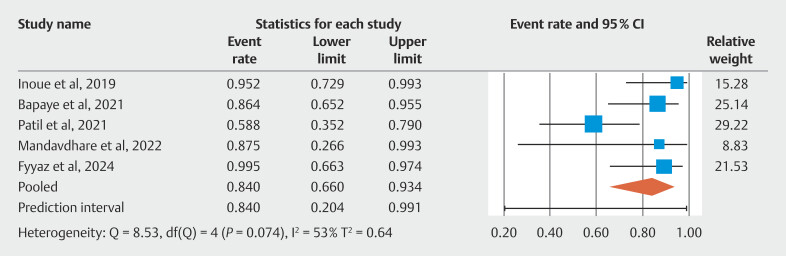
Forest plot showing individual study estimates and pooled estimate for fundoplication wrap-integrity.

**Fig. 5:**
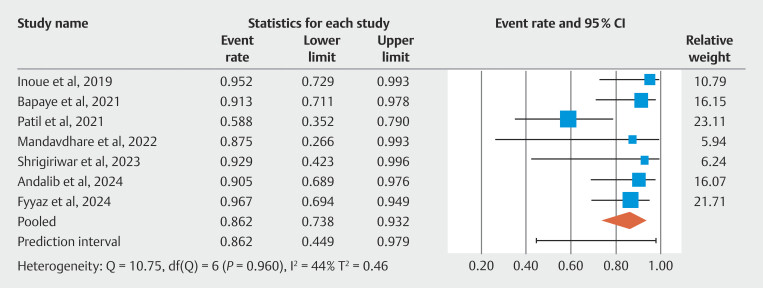
Forest plot showing individual study estimates and pooled estimate for composite outcome of overall clinical success of POEM-F in mitigating post-POEM GER.

All included studies reported data about technical success of POEM and fundoplication. The pooled technical success rate of POEM was 96.90%; 95% CI 91.40–98.90. There was no evidence of heterogeneity calculated using I ^2^ statistic (I ^2^ = 0%). The Begg-Mazumdar bias indicator gave Kendall's tau b value of -0.95 ( *P* = 0.002), suggesting the possibility of publication bias. Likelihood of publication bias could not be ruled out on visual inspection of the funnel plot. The pooled technical success rate of fundoplication was 92.30%; 95% CI 85.20–96.10. The forest plot evaluating the technical success of fundoplication is shown in Fig. 2 . The pooled clinical success rate of POEM in resolving symptoms due to achalasia was 96.40%; 95% CI 90.70–98.60. Fig. 3 shows individual study estimates and the pooled estimate for clinical success of POEM in treating achalasia. Five of seven included studies reported findings on fundoplication wrap integrity on follow-up endoscopy. The pooled rate of intact fundoplication wrap on follow-up was 84.00%; 95% CI 20.4–99.1. There was evidence of moderate heterogeneity calculated using I ^2^ statistic (I ^2^ = 53%). The forest plot for wrap integrity of fundoplication is shown in Fig. 4 . The composite outcome of the overall clinical success of POEM-F in mitigating post-POEM GER was 86.20%; 95% CI 73.80–93.20. There was evidence of moderate heterogeneity calculated using I ^2^ statistic (I ^2^ = 44%). Fig. 5 shows individual study estimates and the pooled estimate for overall clinical success. Abnormal ambulatory pH monitoring to evaluate for post-POEM GER was reported in four studies. The pooled rate of post-POEM GER based on abnormal pH monitoring was 21.80%; 95% CI 10.10–41.00. Endoscopic evidence of esophagitis on follow-up was reported in three studies. The pooled rate of LA grade B, C, or D esophagitis was 14.40%; 95% CI 6.10–33.90.

### Adverse events

The pooled overall AE rate was 19.20%; 95% CI 4.30–55.80, of which the rate of major AEs was 3.60%; 95% CI 1.40–9.40. There was evidence of considerable heterogeneity calculated using I ^2^ statistic (I ^2^ = 82%). All subjects experienced capnoperitoneum during the procedure, which was not considered a complication but an expected part of the procedure and was managed intra-procedurally in all cases. AEs included capnothorax, subcutaneous emphysema, mild post-procedure pain, and asymptomatic eroded clips on follow-up luminal exams.

## Discussion

Symptomatic and asymptomatic GER following POEM is a significant clinical concern and one of the major AEs associated with this procedure. Erosive esophagitis was shown to be more common following POEM than LHM at 3 months (57% vs. 20%) and 24 months (44% vs 29%) in a randomized multicenter study by Werner et al 25 . A meta-analysis comparing 1542 POEM and 2581 LHM patients found symptomatic GERD (19% vs. 8.8%), abnormal pH monitoring (39% vs. 16.8%), and erosive esophagitis (29.4% vs 7.6%) to be more common after POEM than LHM 26 . The American College of Gastroenterology (ACG) and Society of American Gastrointestinal and Endoscopic Surgeons (SAGES) recommended that patients who undergo myotomy have fundoplication to prevent GER 3 4 . However, this is not a common practice following POEM, but it is almost universal following LHM.

Findings from this meta-analysis show that the novel POEM-F procedure, which adds a same-session fundoplication using a pure NOTES approach, has a good technical success rate of about 92%. Reasons for technical failure of fundoplication included unfavorable anatomy, such as a sigmoid esophagus, presence of hiatal hernia, and inability to localize the peritoneal cavity 16 18 . However, the attempt at performing a fundoplication did not impede the POEM procedure because all subjects in whom fundoplication was unsuccessful still had their POEM procedure completed with an overall technical success rate of 97% for POEM. Our findings also add to the literature on efficacy of POEM, which has an excellent clinical success rate of 96% in addressing symptoms due to achalasia. POEM-F offers a significant reduction in incidence of post-POEM GER, with evidence of abnormal distal esophageal acid exposure seen in only about 14% of subjects. This is much lower than the overall rates, even up to 50% reported in current literature 26 . One of the challenges in evaluating the actual clinical implications was that all the available studies are prospective or retrospective case series without a control arm. Furthermore, parameters by which the clinical success of fundoplication and GER were evaluated varied among the included studies.

Documentation of the success of POEM-F in terms of wrap-integrity on follow-up endoscopy was performed in five out of seven studies 14 16 18 19 21 whereas follow-up ambulatory pH monitoring to document presence of significant distal esophageal acid exposure was performed in four studies 16 18 20 21 . Other methods used for GER evaluation included validated questionnaires in two studies 15 16 , need for regular PPI use 15 16 18 , endoscopic evidence of erosive esophagitis 16 18 21 , and measurement of pre-POEM and post-POEM distensibility index at the esophagogastric junction using EndoFLIP 15 21 . Only one study reported a comprehensive evaluation for GER based on the Lyon Consensus, defined as esophageal acid exposure time (EAET) > 6% on pH studies or endoscopic evidence of Grade C or D esophagitis 16 . This study considered LA Grade A or B esophagitis and EAET 4% to 6% as borderline evidence of GER 16 . However, there have been recent updates with Lyon consensus 2.0 establishing LA grade B esophagitis as conclusive evidence of GERD 27 . In our composite outcome assessing the overall clinical success of POEM-F, we incorporated all available endpoints from each study that best aligned with the updated Lyon consensus 2.0 to evaluate for presence of GER.

Mean total procedure time and fundoplication time were 115.74 minutes and 55.28 minutes, respectively. Based on current literature, average procedure time for POEM in proficient endoscopists is about 80 minutes 8 . As is clear from these data, adding fundoplication can add significant time to overall procedure duration. Bapaye et al. noted a significant improvement in total procedure time after the first five procedures (88 vs. 51.2 minutes), suggesting that procedure duration may improve following the initial learning curve 16 . Our study also shows that POEM-F is safe, with an overall major

AE rate of 3.6% and no procedure-related mortality. Bapaye et al. and Patil et al. reported delayed AEs associated with using a clip for fundoplication. The clips were seen eroding onto the luminal side on follow-up endoscopy, although these events had no clinical consequences 16 18 . To overcome potential adverse outcomes associated with leaving foreign bodies in situ, Toshimori et al. suggested modifying the fundoplication process using an endoscopic hand-suturing method that did not involve clips 28 . As more centers gain experience in POEM-F, the procedure may see further refinement, improving efficiency and safety.

There are a few limitations to this study. All the available studies to date are retrospective single-arm and were performed at high-volume expert centers without a control group, which could introduce selection bias. Results seen at centers with acclaimed international experts may not be replicable in the community. Further, the number of subjects in each available study was relatively small, they are from different backgrounds across the world, and the technique of POEM-F was not predefined or standardized. Objective measurement of clinically significant GER in patients with esophageal motility disorder was also not standardized across the studies. This would be important to define patient selection criterion to understand who would benefit from endoscopic fundoplication with POEM. These factors can influence generalizability of our findings and introduce heterogeneity. Another limitation was the relatively short follow-up duration of less than 1 year. A high-quality randomized controlled trial with a standard, evidence-based definition to assess for clinically significant post-POEM GER in patients with esophageal motility disorders is warranted.

## Conclusions

POEM-F is an effective procedure in expert hands with good technical success and an acceptable AE rate. It appears to offer clinical benefit for mitigating post-POEM GER. However, further standardization for evaluating clinically significant post-POEM GER and the long-term benefit of POEM-F is warranted.

## Notes

Correction **Peroral endoscopic myotomy with fundoplication (POEM-F) for achalasia: Systematic review and meta-analysis** Harishankar Gopakumar, Eugene Annor, Ishaan Vohra et al. Endoscopy International Open 2025; 13: a25368132. DOI: 10.1055/a-2536-8132 In the above-mentioned article institution 9 was corrected. This was corrected in the online version on 20.03.2025.
