# Correction: Peroral endoscopic myotomy with fundoplication (POEM-F) for achalasia: Systematic review and meta-analysis

**DOI:** 10.1055/a-2562-4787

**Published:** 2025-03-19

**Authors:** Harishankar Gopakumar, Eugene Annor, Ishaan Vohra, Iman Andalib, Amy Tyberg, Avik Sarkar, Haroon Shahid, Mine Carames, Juan Carlos Carames, Giovanna Porfilio Gularte, Abed Al-Lehibi, Resheed Alkhiari, Amol Bapaye, Carlos Robles-Medranda, Michel Kahaleh

**Affiliations:** 1 24490 Gastroenterology and Hepatology, OSF Saint Joseph Medical Center, Bloomington, United States; 2 17120 Department of Internal Medicine, University of Illinois Chicago College of Medicine at Peoria, Peoria, United States; 3 17120 Department of Gastroenterology, University of Illinois Chicago College of Medicine at Peoria, Peoria, United States; 4 3673 Gastroenterology, Hackensack Meridian Hackensack University Medical Center, Hackensack, United States; 5 24263 Gastroenterology & Hepatology, Hackensack Meridian JFK University Medical Center, Edison, United States; 6 Gastroenterology, Santander Hospital, Bucaramanga, Colombia; 7 Gastroenterology, Instituto Misionero de Gastroenterología y Motilidad Digestiva, Posadas, Argentina; 8 37849 Gastroenterology and Hepatology, King Fahad Medical City, Riyadh, Saudi Arabia; 9 48032 Division of Gastroenterology, Department of Medicine, College of Medicine, King Saud University, Riyadh, Saudi Arabia; 10 Shivanand Desai Center for Digestive Disorders, Deenanath Mangeshkar Hospital and Research Center, Pune, India; 11 Gastroenterology, Instituto Ecuatoriano de Enfermedades Digestivas - IECED, Guayaquil, Ecuador; 12 Endoscopy, Omni Hospital, Guayaquil, Ecuador; 13 Gastroenterology, Foundation of Interventional and Therapeutic Endoscopy, New Brunswick, United States



In the above-mentioned article institution 9 was corrected. This was corrected in the online version on 20.03.2025.

